# Characteristics and outcomes among a hospitalized patient cohort with *Streptococcus pneumoniae* infection

**DOI:** 10.1097/MD.0000000000020145

**Published:** 2020-05-01

**Authors:** Scott T. Micek, James Simmons, Nicholas Hampton, Marin H. Kollef

**Affiliations:** a Department of Pharmacy Practice, St. Louis College of Pharmacy; b Center for Clinical Excellence, BJC Healthcare; c Division of Pulmonary and Critical Care Medicine, Washington University School of Medicine, St. Louis, MO, USA.

**Keywords:** antibiotic resistance, clinical outcomes, septic shock, streptococcus pneumoniae

## Abstract

Infection due to *Streptococcus pneumoniae* (SP) requiring hospitalization is common. However, recent clinical studies describing patient characteristics and outcomes for SP infection in adults requiring hospitalization are lacking. Our goal was to evaluate patient characteristics, contemporary antibiotic resistance, and clinical outcomes among hospitalized adults with SP infections.

A retrospective cohort study was conducted at Barnes-Jewish Hospital (1350 beds) in St. Louis, Missouri, USA for years 2012 through 2016. During the study period, 358 hospitalized adults, excluding those with meningitis, were identified with SP infection. Forty-four patients (12.3%) died within 30 days of the identification of their infection. Among these infections, 99 (27.7%) were assessed to be hospital-acquired and 259 (72.3%) were community-onset infections. The majority of infections involved the respiratory tract (88.5%). Azithromycin resistance was the most common antibiotic resistance at 51.4%, followed by enteral penicillin resistance (45.3%), trimethoprim-sulfamethoxazole (34.1%), second-generation cephalosporin (cefuroxime) (30.7%), and meropenem (22.6%). There were 70 isolates (19.6%) classified as multidrug resistant. Independent predictors of hospital mortality included increasing weight in 1-kilogram increments (adjusted odds ratio [AOR], 1.02; 95% CI, 1.01 – 1.02; *P* = .048), increasing Charlson Comorbidity Index scores (AOR, 1.31; 95% CI, 1.21 – 1.42; *P* = .001), and the presence of septic shock (AOR, 3.89; 95% CI, 2.31 – 6.57; *P* = .009). The median [interquartile range] hospital length of stay was 8.1 days [4.5 days, 16.8 days].

Hospitalized patients with infection attributed to SP have significant 30-day mortality and use of hospital resources. Antibiotic resistance is common among isolates associated with infection. Determinants of mortality are primarily severity of illness, underlying comorbidities and increasing patient weight. Efforts to improve the treatment and prevention of SP infections are needed.

## 1 Introduction

*Streptococcus pneumoniae* (SP) is a common cause of community-onset infection.^[1]^ The ability of SP to readily colonize the mucosal surfaces of the upper airways and evade host inflammatory and immune responses contributes to its transmission and virulence.^[2]^ Pneumococcal diseases range from mild respiratory tract mucosal infections such as otitis media and sinusitis to diseases of greater severity including meningitis, pneumonia and septic shock.^[3]^ The Centers for Disease Control and Prevention and other sources report that there are up to 60,000 cases of invasive pneumococcal infection each year, with at least 40% of the clinical isolates being resistant to at least 1 antibiotic commonly employed for empiric treatment of SP infections.^[4,5]^ However, up to 30% of SP isolates may be resistant to more than 1 antimicrobial agent.^[5,6]^

Macrolide antibiotics are still employed as a treatment option for infections attributed to SP depending on the prevailing resistance rates.^[7,8]^ Yet, rates of resistance to macrolides are reported to be between 20% and 40%.^[9,10]^ Several recent reports suggest that macrolide resistance is much higher in specific regions of the world with China recording greater than 90% resistance in some areas.^[11,12]^ The high rates of resistance to macrolides are in large part related to the escalating global consumption of macrolide antibiotics.^[13–15]^ Given the rising rates of macrolide resistance, and resistance of pneumococci to other antibiotic classes, new antibiotics have been developed for the treatment of antibiotic resistant SP infections.^[16,17]^

Due to the importance of SP as a cause of invasive infection, we carried out a retrospective study with 2 main goals:

(1) to determine the contemporary rates of SP resistance to antibiotics commonly employed to treat SP infections; and
(2) to assess the outcomes of infection with SP among hospitalized adults.

Given the aging and escalating rates of immune suppression among both ambulatory individuals and hospitalized patients, the numbers and severity of SP infections is expected to grow in the future.^[18]^

## 2 Methods

### 2.1 Study population and data source

The study was conducted within Barnes-Jewish Hospital, an academic referral center of 1350 beds. Washington University School of Medicine Human Studies Committee (Institutional Review Board # 201801189) and the St. Louis College of Pharmacy (Institutional Review Board #2018–30) approved this study and waived the need for informed consent. All patients with a microbiologically confirmed SP infection treated with antibiotics from January 1, 2012 through December 31, 2016 were eligible for inclusion with the exception of patients with meningitis. SP meningitis was excluded due to the limited numbers typically seen at our institution and the difference in disease severity compared to other forms of SP infection. All data was derived from the informatics database provided by the Center for Clinical Excellence, BJC HealthCare, St. Louis, Missouri.

### 2.2 Study outcomes/objectives

The primary objective of this study was to determine the contemporary rates of SP resistance to antibiotics commonly employed to treat infections attributed to Gram-positive cocci. The secondary objective of this study was to assess the outcomes of infection with SP among hospitalized adults including 30-day mortality and hospital length of stay.

### 2.3 Definitions and study design

SP infection was defined by the microbiologic isolation of SP from sterile sites (blood, pleural fluid, synovial fluid, ascites, bone) or from skin and soft tissue sites or respiratory specimens in the context of clinical features suggesting the presence of active infection. For skin and soft tissue infections medical record documentation of the presence of purulence or cellulitis was required. For respiratory infections clinical criteria in line with the American Thoracic Society position statement on pneumonia were required.^[19]^ These diagnostic criteria included presence of a new or progressive radiographic infiltrate and at least 2 of the following clinical features: fever >38°C, leukocytosis (>10 × 10^9^cells/L), leukopenia (≤4 × 10^9^cells/L), or purulent respiratory secretions. The presence of a new or progressive radiographic infiltrate was based on the interpretation of the chest radiograph by board-certified radiologists.

Septic shock was defined as the need for vasopressors (norepinephrine, dopamine, vasopressin, epinephrine, or phenylephrine). Only the first episode of documented SP infection was recorded. Antimicrobial treatment was classified as initially appropriate antibiotic therapy if the initial regimen had in vitro activity demonstrated against the SP isolate. Multidrug-resistant isolates had to demonstrate in vitro resistance to at least 3 distinct classes of antimicrobials that would normally have activity against SP.^[20]^

### 2.4 Antimicrobial susceptibility testing

The microbiology laboratory performed antimicrobial susceptibility testing of the bacterial isolates using the MICroSTREP Plus minimum inhibitory concentration panel (Beckman Coulter, Brea, California), with breakpoints established by the Clinical Laboratory and Standards Institute published during the inclusive years of the study.^[21]^ For example, the breakpoint for resistance to penicillin is greater than 2 mg/L.^[21]^ All classifications of antibiotic resistance were based on in vitro susceptibility testing using these established breakpoints.

### 2.5 Statistical analyses

The sample size was determined by a convenience sample of all patients with SP infection identified in the hospital during the study period. Continuous variables were expressed as mean and standard deviation or median and interquartile range when appropriate. The *t* test was used to analyze normally distributed continuous variables, whereas the Mann–Whitney *U* test was used to analyze non-normally distributed continuous variables. Categorical data were reported as frequency distributions and analyzed using the chi-square or Fisher exact tests. *P* values less than .05 were considered statistically significant, and all tests were 2-tailed. We performed multivariate logistic regression analysis to identify risk factors associated with 30-day mortality. All risk factors that were significant at the 0.20 level in the univariate analyses were included in the corresponding multivariable analysis. All variables entered into the model were examined for colinearity using the variance inflation factor as a colinearity statistic. The model's goodness of fit was assessed via determination of the Hosmer-Lemeshow c-statistic. A sensitivity analysis was also planned for the pneumonia subpopulation. All analyses were done using SPSS Statistics 24 (IBM SPSS Statistics, Version 24.0. Armonk, NY).

## 3 Results

During the study period, 358 patients excluding those with meningitis were identified with monobacterial SP infection during their hospitalization. Azithromycin resistance was the most common antibiotic resistance observed at 51.4%, followed by enteral penicillin resistance (45.3%), trimethoprim-sulfamethoxazole (34.1%), second-generation cephalosporin (cefuroxime) (30.7%), and meropenem (22.6%) (Table 1). There were 70 isolates (19.6%) classified as multidrug resistant. Thirty-day nonsurvivors had greater resistance to second generation cephalosporins, meropenem, and penicillin compared to 30-day survivors (Table 1). Multidrug resistance and resistance to second- and third-generation cephalosporins, meropenem, amoxicillin-clavulanate, macrolides, tetracycline, clindamycin, and trimethoprim sulfamethoxazole was statistically more common among the non-bacteremic isolates. Hospital-acquired isolates were statistically more likely to be resistant to second-generation cephalosporins and clindamycin compared to community-acquired solates.

**Table 1:**
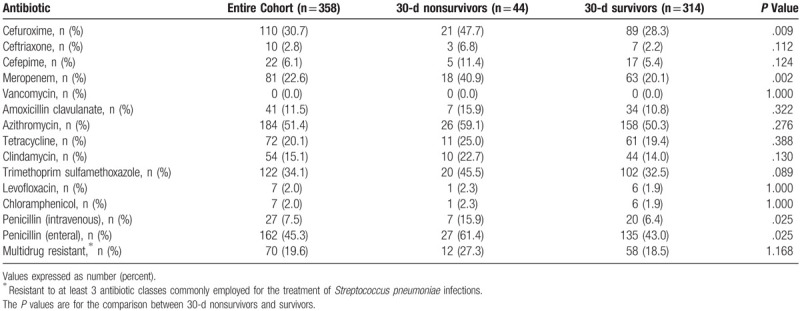
Antibiotic resistance of the isolates.

The mean age of patients was 55.6 ± 15.1 years and the median [interquartile range] for acute physiology and chronic health evaluation II scores was 11 [7.0,15.3] (Table 2). SP infection was more common among males (60.1%). Underlying chronic obstructive pulmonary disease existed in 42.7% and active malignancy was present in 29.6% of patients. The most common infection was pneumonia (88.5%) (Table 3). Positive blood cultures for SP were present in 33.2% of patients and forty-four patients (12.3%) died within 30 days of the identification of their infection (Tables 3 and 4). Among these infections, 99 (27.7%) were assessed to be hospital-acquired with 259 (72.3%) being community-onset infections (Table 4). The median [interquartile range] hospital length of stay was 8.1 days [4.5 days, 16.8 days].

**Table 2:**
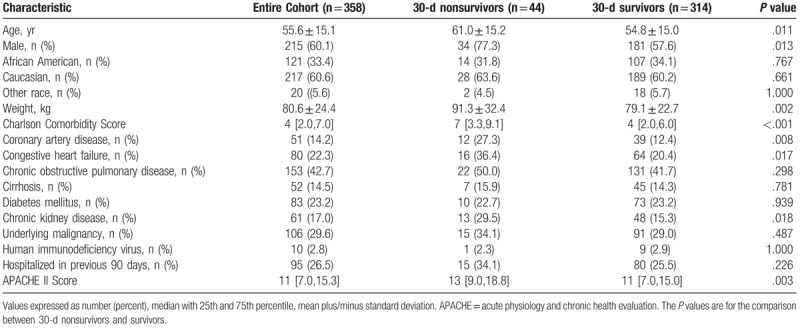
Baseline characteristics.

**Table 3:**
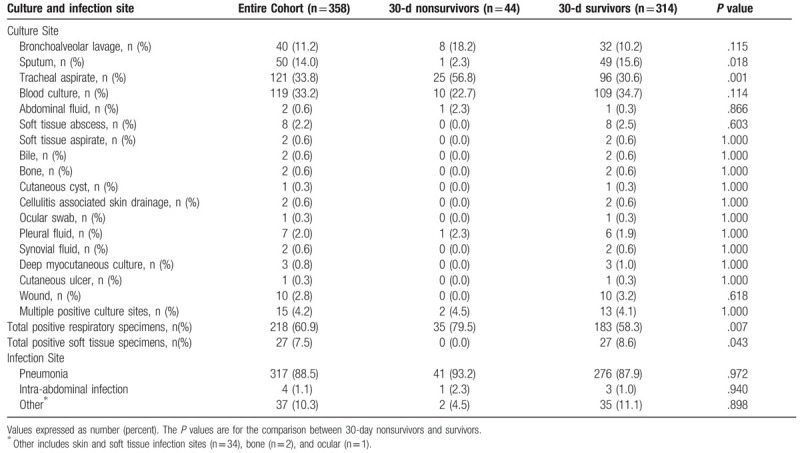
Positive microbiologic cultures and infection site.

**Table 4:**
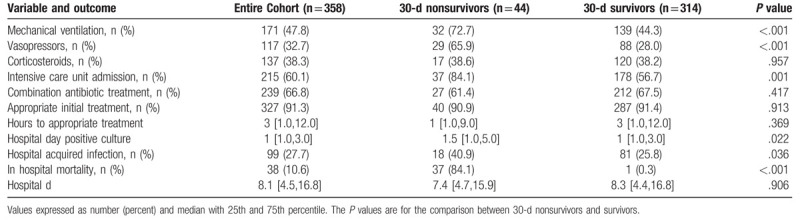
Process of care variables and outcomes.

Thirty-day nonsurvivors were significantly older, more likely to be male, had greater weight, Charlson Comorbidity Index scores, coronary artery disease, congestive heart failure, chronic kidney disease and greater Acute Physiology and Chronic Health Evaluation II scores compared to 30-day survivors (Table 2). Sputum cultures were statistically more common for identifying SP among 30-day survivors while tracheal aspirate cultures were statistically more common for identifying SP among 30-day nonsurvivors (Table 3). Thirty-day nonsurvivors more frequently required mechanical ventilation, the use of vasopressors, admission to the ICU setting, and were significantly more likely to have a hospital-acquired infection (Table 4). Hospital duration was similar between 30-day nonsurvivors and 30-day survivors.

**Figure 1:**
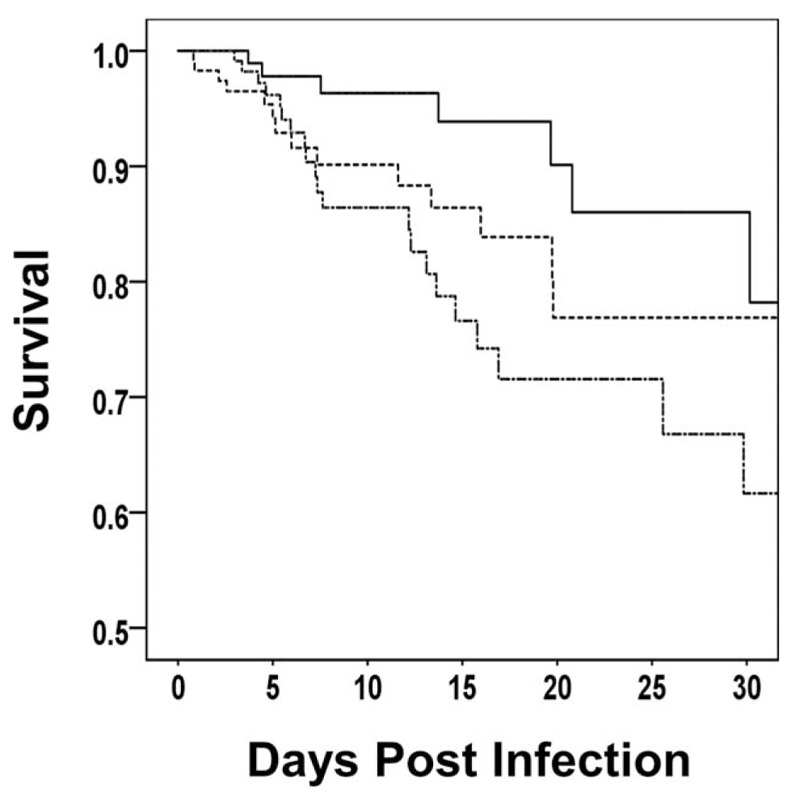
Kaplan-Meier survival curves depicting the tertiles of body weight among patients with *Streptococcus pneumoniae* infection. Tertile weight range: lowest tertile, 34.1 kg to 67.6 kg; intermediate tertile, 68.0 kg to 86.7 kg; highest tertile, 87.0 kg to 206.9 kg. The solid line shows the patients within the lowest weight tertile. The dashed line represents the patients within the intermediate weight tertile. The dotdashed line shows the patients within the highest weight tertile. (Tarone-Ware statistic, *P* = .044.).

The average patient weight of our study population was 80.6 ± 24.4 kg. Figure 1 shows that survival post SP infection was negatively associated with increasing weight. Patients within the lowest weight tertile had the greatest survival while those within the greatest weight tertile had the lowest survival from SP infection. Logistic regression analysis identified independent predictors of 30-day mortality as increasing weight in 1-kilogram increments (adjusted odds ratio [AOR], 1.02; 95% CI, 1.01 – 1.02; *P* = .048), increasing Charlson Comorbidity Index scores (AOR, 1.31; 95% CI, 1.21 – 1.42; *P* = .001), and the presence of septic shock (AOR, 3.89; 95% CI, 2.31 – 6.57; *P* = .009) (Hosmer-Lemeshow test, *P* = .473) (Table 5). Repeating the logistic regression analysis for the 317 (88.5%) patient with pneumonia yielded similar independent predictors for 30-day mortality: increasing weight in 1-kilogram increments (AOR, 1.02; 95% CI, 1.01 – 1.02; *P* = .014), increasing Charlson Comorbidity Index scores (AOR, 1.25; 95% CI, 1.18 – 1.32; *P* < .001), and the presence of septic shock (AOR, 4.41; 95% CI, 3.09 – 6.32; *P* < .001) (Hosmer-Lemeshow test, *P* = .996).

**Table 5:**
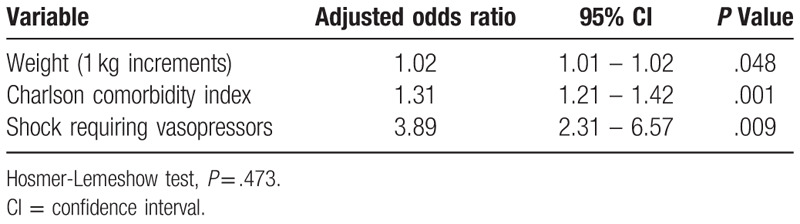
Independent Predictors of Hospital Mortality.

## 4 Discussion

We found that more than 50% of the SP clinical isolates associated with infection among hospitalized adults were resistant to azithromycin. Moreover, almost 20% of all SP isolates were classified as multidrug resistant. Overall thirty-day mortality was 12.3% and greater than a quarter of the infections were hospital acquired. Independent risk factors for 30-day mortality included increasing weight, greater comorbidities as assessed by the Charlson Comorbidity Index score and the presence of septic shock requiring vasopressor administration. Initially appropriate antibiotic therapy was delivered in more than 90% of patients despite the high rate of antibiotic resistance. This was likely due to the antibiotic treatment protocols at Barnes-Jewish Hospital recommending inpatient treatment with a third generation cephalosporin antibiotic or broader, often in combination with other agents, for patients with serious infections admitted from the community setting or developing hospital-acquired infections as outlined in the Barnes-Jewish Hospital Tool Book.^[22]^

Antibiotic resistance among SP isolates has been increasing steadily in the United States as reported by several groups including the Center for Disease Control and Prevention.^[4,9,10,23]^ Unfortunately, most invasive pneumococcal disease primarily affects young children, adults over the age of 65 years, and individuals with underlying immune suppression and significant comorbidities.^[24]^ Our study demonstrated high rates of antibiotic resistance among hospitalized adults with pneumococcal infections. This finding is in line with the report by Keedy and associates presented at the 2016 ID Week meeting showing that 33% of all SP isolates demonstrated high level resistance to macrolides.^[25]^ An important aspect of our study, compared to other investigations that have focused on the outpatient setting where oral agents such as macrolides are commonly employed, is that we exclusively evaluated a hospitalized population.^[26,27]^ However, it is worth noting that in some cases, lack of outpatient treatment with an appropriate agent active against the SP isolate has led to life-threatening disease including the need for extracorporeal membrane oxygenation.^[28]^

A unique finding of our study was the association between increasing patient weight and 30-day mortality. Several studies in children or adults suggest that both underweight and obesity are associated with increased infection risk.^[29]^ However, confounding factors such as malnutrition, hygienic status and underlying disease or co-morbidities might aggravate accurate assessment of the impact of body weight on infection risk. Obesity specifically is thought to primarily increase overall mortality by escalating certain disease processes such as diabetes mellitus, coronary artery disease, cancer, and renal disease.^[30]^

Obesity is also thought to negatively influence immune function and host defense mechanisms. One potential pathway for this influence is that obesity is an inflammatory condition associated with chronic activation of the immune system and consequent local and systemic inflammation.^[31]^ This state of chronic inflammation, associated with other influences of obesity such as diabetes, can negatively impact the immune system by reducing immune responses resulting in an increased risk of infection.^[31]^ Moreover, obesity can induce a state of metaflammation in which excess nutrients, due to inefficient glucose metabolism, promote chronic low-grade inflammation the hallmarks of which are high levels of glucose, lipids, free fatty acids, and reactive oxygen species.^[32]^ Frasca et al also recently suggested that obesity could increase the prevalence of pneumococcal disease, and potentially worsen the outcome of infected patients, due to the combined effects of increased comorbidities such as diabetes in conjunction with the reduced immune response promoted by chronic inflammation.^[33]^

There are several important limitations of our study. First, these data were obtained from a single urban medical center. It is possible that our findings may differ from those obtained at other centers due to differences in patient case mix and disease severity. The high rate of hospital-acquired SP infections in our cohort is likely a reflection of the overall disease mix of our patient population as previously demonstrated.^[34]^ Second, we employed patient weight and not the body mass index due to the lack of patient height data in the electronic medical record. This may have influenced our finding of a relationship between weight and 30-day mortality. However, other studies have demonstrated that body mass index may not be an accurate method for determining the presence of obesity suggesting that body weight is an appropriate marker for study purposes.^[35,36]^ Third, we did not collect outpatient data including prior antibiotic therapy. This may be an important contributor to patient outcomes that our study way not able to account for. Fourth, the sample size of our study population may have limited our ability to identify all of the important risk factors for 30-day mortality. Finally, we did not assess the individual SP isolates to determine whether specific virulence factors may have contributed to the observed patient outcomes.^[37]^

In conclusion, our study demonstrates that hospitalized patients with SP infection have significant 30-day mortality and use of hospital resources. Antibiotic resistance, especially to macrolides and second-generation cephalosporins, is common among SP isolates associated with infection. Determinants of mortality for SP infections are primarily severity of illness, comorbidities and increasing patient weight. Efforts aimed at improving the treatment and prevention of infections caused by SP are required. Such efforts may include targeted vaccination programs for patients at high risk for developing SP infection including patients with obesity and the use of macrolide antibiotics as adjunctive treatment when a high systemic inflammatory response is present .^[38,39]^ The latter has been the rationale for including macrolides as part of the treatment regimen for SP infections even when resistance is demonstrated.^[40]^ Additional investigations are warranted to verify our findings and to determine the optimal treatment approaches for SP infections. Developing new prevention strategies and treatment paradigms for SP infections is critical given the lack of mortality improvement with this infection over the past twenty years.^[41]^

## Author contributions

STM, JS, NH, MHK each contributed to the study design, data collection, data analysis, manuscript draft formulation, and approval of the final manuscript version.
